# Improving patient safety through identifying barriers to reporting medication administration errors among nurses: an integrative review

**DOI:** 10.1186/s12913-021-07187-5

**Published:** 2021-10-25

**Authors:** Agani Afaya, Kennedy Diema Konlan, Hyunok Kim Do

**Affiliations:** 1 College of Nursing, Yonsei University, 50-1, Yonsei-ro, Seodaemun-gu, Seoul, 03722 South Korea; 2 School of Nursing and Midwifery, University of Health and Allied Sciences, Ho, Ghana

**Keywords:** Barriers, Integrative review, Medication administration error, Nurses

## Abstract

**Background:**

The aim of the third WHO challenge released in 2017 was to attain a global commitment to lessen the severity and to prevent medication-related harm by 50% within the next five years. To achieve this goal, comprehensive identification of barriers to reporting medication errors is imperative.

**Objective:**

This review systematically identified and examined the barriers hindering nurses from reporting medication administration errors in the hospital setting.

**Design:**

An integrative review.

**Review methods:**

PubMed, Web of Science, EMBASE, and the Cumulative Index to Nursing and Allied Health Literature (CINAHL) including Google scholar were searched to identify published studies on barriers to medication administration error reporting from January 2016 to December 2020. Two reviewers (AA, and KDK) independently assessed the quality of all the included studies using the Mixed Methods Appraisal Tool (MMAT) version 2018.

**Results:**

Of the 10, 929 articles retrieved, 14 studies were included in this study. The main themes and subthemes identified as barriers to reporting medication administration errors after the integration of results from qualitative and quantitative studies were: organisational barriers (inadequate reporting systems, management behaviour, and unclear definition of medication error), and professional and individual barriers (fear of management/colleagues/lawsuit, individual reasons, and inadequate knowledge of errors).

**Conclusion:**

Providing an enabling environment void of punitive measures and blame culture is imperious for nurses to report medication administration errors. Policymakers, managers, and nurses should agree on a uniform definition of what constitutes medication error to enhance nurses’ ability to report medication administration errors.

**Supplementary Information:**

The online version contains supplementary material available at 10.1186/s12913-021-07187-5.

## Introduction

Improving patient safety remains an ongoing global health challenge for more than two decades after the beginning of the new wave of attention by the United States (US) Institute of Medicine (IOM) in 1999 report “To err is human” [1–4]. In March 2017, the World Health Organisation (WHO), released an article called “Medication Without Harm, WHO Global Patient Safety Challenge”, to gear up the process of change to reduce the impact of patient harm associated with unsafe medication practices by health care practitioners [5]. The aim of the third WHO challenge released in 2017 was to attain a global commitment, involvement, and prevention strategies to lessen the severity and to prevent medication-related harm by 50% within the next five years [5–7]. One of the ten leading causes of disability and deaths in the world is the occurrence of adverse events arising due to errors [8]. In developed countries, approximately one in every ten patients suffers harm while receiving care [9, 10] in the hospital with 50% of them being preventable [8]. It is also estimated that each year, 134 million adverse effects occur in hospitals within developing countries resulting in 2.6 million deaths due to unsafe care [8].

Medication error (ME) reporting systems represent a central tool for retrospective medication safety risk management in many healthcare organisations as they provide information on the occurred incidents [11]. However, these systems may become worse if adequate measures are not taken to ensure an enabling environment in reporting MEs [12]. Nurses are the most significant healthcare workforce in the healthcare sector, primary caregivers, and play a vital role in the prevention and detection of adverse events in patients [4]. Their roles in reporting medication administration errors (MAEs) are pivotal because they are directly involved in the administration of the vast majority of the medications ordered in hospitals [13].

MEs are the leading causes of avoidable patient harm in the health care system across the world [14] and nurses are among the biggest contributors to MEs [15]. Al-Worafi [16] revealed that 39% of MEs occur among general practitioners, 38% among nurses, and 23% among pharmacists. Also, Ferrah, Lovell, and Ibrahim [17], in their systematic review indicated that the prevalence of MEs among nurses is between 16 and 27%. These figures are worrying and therefore nurses reporting MAEs in the health care system will enhance root cause analysis. This will lead to the identification of the specific causes of MEs and therefore provide concrete solutions to reduce medication harm to patients. It is also essential for nurses to report MEs because nurses represent the last safety check in the chain of events in the drug administration process, and are the final safeguard of patient wellbeing [14].

### Objective

This review systematically identified and examined the barriers hindering nurses from reporting medication administration errors in the hospital setting.

## Methods

An integrative review method based on Whittemore and Knafl’s [18] methodological approach was employed to identify primary studies that focused on barriers to reporting medication administration errors (MAEs) among nurses. Unlike the traditional systematic review, an integrative review utilises a broad focus and allows for the analysis of diverse data sources (qualitative, quantitative, and mixed-method studies) [18] to inform research and practice. Whittemore and Knafl’s approach strengthens the rigor of an integrative review of nursing evidence and plays a vital role in the development of evidence-based healthcare initiatives. The study was guided by the five steps of Whittemore and Knafl’s which fostered a thorough methodological approach focusing on problem identification, literature search, data evaluation, data analysis, and presentation of study characteristics [18]. The first step focused on why this review is essential. The second step detailed how the reviewers conducted a robust literature search using the Preferred Reporting Items for Systematic reviews and Meta-Analysis guidelines (PRISMA). The third step detailed how the articles were assessed for rigor using the Mixed Method Appraisal Tool (MMAT) version 2018 [19]. The last step involved data analysis and presentation of findings from the reviewed articles.

### Problem identification

The researchers observed that nurses’ inability to report MAEs is hindered by multiple organisational and individual barriers. Several studies have identified some organisational and individual barriers to reporting MAEs such as; lack of reporting systems, blaming individuals instead of the system, no feedback after reporting MAE, negative response from reporting MAEs, lack of clear definition for ME, and fear of reprimand and punishment [20–22]. Therefore, the need to systematically synthesize current available studies from a wider international perspective to inform nurses and policymakers on strategies to improve MAE reporting and the prevention of patient harm in health facilities.

### Literature search

The Preferred Reporting Items for Systematic Reviews and Meta-Analyses (PRISMA) framework was used for the identification and screening of articles [23, 24]. A search of electronic databases (PubMed, Web of Science, EMBASE, and the Cumulative Index to Nursing and Allied Health Literature (CINAHL)) identified articles published between January 2016 to December 2020. To determine the search parameters, the Population, Intervention, Comparison, Outcome (PICO) framework was used. Nurses were the population for this review, the intervention was reporting MAE, there was no comparison, and the outcome was barriers to reporting MAEs. The following keywords and combinations were used: medication error*/medicine error*/drug error*; report*/disclosure; nurs*. The inclusion and exclusion criteria for this review is shown in Table 1.

**Table 1:** Inclusion and exclusion criteria

**Inclusion criteria**	
Topic	• The main focus is on barriers to reporting MAEs among nurses
Population	• Practicing nurses at the hospital setting
Types of study	• Quantitative, qualitative, and mixed-method studies
Language	• English
Times frame	• Studies published within January 2016 to December 2020
**Exclusion**	
	• Studies focused on barriers to reporting MEs among other health professionals but not nurses. Studies focused on nursing errors, review articles, discussions, editorial, conference papers, notes, commentary pieces, books, abstracts, and duplicates.

### Quality appraisal

Two researchers (AA and KDK) independently assessed the quality of all the included studies using the MMAT version 2018 [25]. Disagreements between the two researchers (AA and KDK) were discussed and a consensus was built with HKD. The MMAT contains methodological quality criteria for appraising qualitative, quantitative, and mixed methods studies. The MMAT evaluates the appropriateness of the study aim, study design, methodology, recruitment of participants, data collection, analysis of data, presentation of results, discussions by authors, and conclusions. The studies were rated as high, moderate, and low in quality. The researchers did not assign the overall quality score as it is discouraged by Hong et al. [25] but the methodological quality of the studies were assessed in accordance to the guideline provided by Hong et al. [25].

### Data extraction and synthesis

For data extraction, a matrix was developed to extract relevant information from the studies which included information about the authors, study aim, study design, sample size and characteristics, key findings concerning barriers to reporting MAEs among nurses. Two researchers (AA and KDK) were involved in data extraction. Disagreements were resolved through discussion with the third author (HKD). A convergent synthesis design was adopted to integrate results from qualitative, quantitative, and mixed-method studies and transformed them into qualitative findings [26]. A thematic approach was used to synthesize key findings emerging from included articles in relation to barriers to reporting MAEs among nurses, which were read thoroughly and coded by two of the researchers (AA and KDK). The codes were reviewed, and similar codes were categorized to form descriptive themes. The descriptive themes were assessed to generate meaning beyond the original data leading to the development of new, interpretive analytical themes. The researchers (AA and KDK) synthesized the data independently, discrepancies were discussed (AA, KDK, and HKD), and a consensus was built before finalizing the overarching themes and subthemes.

## Results

### Study selection

**Fig. 1:**
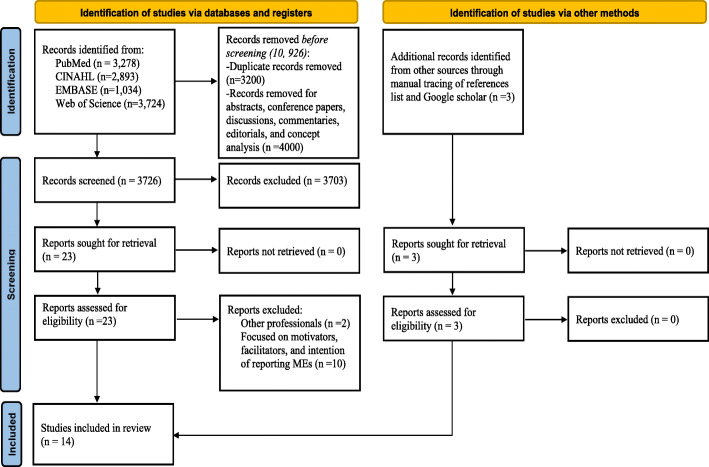
PRISMA 2020 flow diagram

The search of all the electronic databases yielded 10,926 articles. Citations for the articles were imported into Endnote X9 (version 1.19.6) reference manager for screening, removal of duplicates, and storage. Additional articles (*n* = 3) were searched from Google Scholar and through manually tracing of relevant literature from the list of references in the included studies. A total of 3726 non-duplicate articles were screened by title and abstract using the standard integrative review process (inclusion and exclusion criteria) (Table 1). Following the title and abstract screening, 23 articles were included. Of the remaining 23 sources, 12 articles were excluded following a full-text review. Discrepancies were resolved through discussions and the final articles were agreed on. In addition to the 3 additional articles retrieved from manual tracing of reference list and Google Scholar, finally, a total of 14 studies were included in the review. Figure 1 illustrates the PRISMA flow diagram.

### Study characteristics

This review was based on 14 peer-reviewed original publications on barriers to reporting MAEs by nurses in different countries. The study approaches used mainly quantitative descriptive cross-sectional [12], mixed-method [1], and qualitative study explorative design [1]. The cumulative sample size comprised 3299 nurses. The sample size for the quantitative studies ranged from 135 to 548 and the qualitative study involved 23 nurses. Three studies were conducted in Iran [27–29] and Saudi Arabia [13, 30, 31], and a study each in Malaysia [32], Jordan [33], South Korea [34], Taiwan [35], United States [36], Ethiopia [37], Pakistan [38] and Turkey [39]. Two studies utilized a theoretical or conceptual framework. The Theoretical Domains Framework model was utilized by Alrabadi et al. [33], and the Theory of Planned Behaviour was utilized by Shahzadi et al. [38] (See Table 2).

**Table 2:** Summary of study findings

First Author/year	Country	Aim of study	Design	participants	Key findings (Barriers)
Alrabadi (2020) [33]	Jordan	To explore nurses’ understanding, perception, attitude, and prevalence of MEs and thereafter defining the main factors associated with its occurrence and needed for designing proper policies for its sufficient prevention.	Cross-sectional study design	156 nurses	Underreporting was related to fear of losing a job. Nurses not acknowledging the gravity of the MEs to necessitate reporting. Fear of colleague nurses’ actions. Nurses’ knowledge about what constitutes ME. The use of medication incident reporting was a barrier to underreporting of MEs.
Lee (2017) [34]	South Korea	To identify differences in what nurses, consider as MAEs, to examine their willingness to report these errors, and to identify barriers to reporting MEs by hospital type.	Cross-sectional, study design	548 nurses	Fear of negative consequences was a major barrier to ME reporting. Fear of legal actions against nurses by patients or their families. ME reporting consumed much time. Fear of criticism from colleagues or other professionals was also a barrier to ME reporting. Fear of managers’ reactions or punitive measures against nurses. No feedback is given after reporting MAEs.
Alamrani, (2020) [30]	Saudi Arabia	To investigate barriers to MAEs reporting and to identify the reasons for MEs among nurses in Saudi Arabia.	Cross-sectional study design	321 nurses	Nursing administration focuses on the individual rather than using the systems approach to solve the problems. Lack of feedback from authorities. Nurses felt they could be blamed if something negative happened to the patient. Much emphasis is placed on MEs as a measure of the quality of nursing care. Nurses feared negative consequences from reporting MAEs. ME is not clearly defined. Nurses did not think the error was important enough to report. Disagreement with the hospital’s definition of a ME. Nurses were unaware of the occurrence of MAEs.
Dyab et al. (2018) [32]	Malaysia	To explore nurses’ knowledge on ME reporting by determining their attitudes towards reporting and studying the implicated barriers and facilitators.	Exploratory qualitative design	23 nurses	Lack of time to report MEs. Tiredness and heavy workload. Nurses felt they would be embarrassed if they reported MAEs. Fear of being blamed. Fear of punitive actions/investigations. Fear of negative impact on job records. Lack of confidentiality in the reporting system. No feedback on previously reported MEs.
Yung et al. (2016) [35]	Taiwan	To explore the attitudes and perceived barriers to reporting MEs and to understand the characteristics of – and nurses’ feelings – about error reports.	Cross-sectional study design	306 nurses	Nurses with no reporting experience. MAE occurrence without patient harm. Nurses who could not identify errors did not report. Fear of blame from superiors. Fear of being labelled as incompetent and inadequate nurses.
Nourian et al. (2020) [27]	Iran	aimed to determine the barriers of reporting MAEs from the point of view of nurses in neonatal and neonatal intensive care units.	Cross-sectional study design	157 nurses	Fear of legal action by patient or relatives. Afraid of the adverse consequences of reporting MEs. No positive feedback is given for passing medications correctly. Nursing administration focuses on the individual rather than looking at the systems as a potential cause of the error.
Bifftu et al. (2016) [37]	Ethiopia	This study aimed to assess the prevalence of ME reporting and associated factors among nurses working at The University of Gondar Referral Hospital, Northwest Ethiopia	Cross-sectional study design	282 Nurses	Level of education. Disagreement overtime error definition. Fear of consequence and for administrative reasons.
Shahzadi et al. (2017) [38]	Pakistan	To assess the barrier in reporting MAEs among nurses.	Cross-sectional study design	222 Nurses	Nurses did not recognize ME. Nurses did not take MEs to be significant. Reporting takes much time. Negative response from the hospital administration. No proper ME reporting system.
Abdullah et al. (2017) [28]	Iraq	1. To assess the causes of medication errors. 2. To assess the barriers that prevent nurses from reporting MEs. 3. To find out the association between nurses’ demographic data and causes of MEs. 4. To find out the association between nurses’ demographic data and barriers to reporting MEs.	Cross-sectional study design	150 Nurses	Negative attitude toward the nurse by either patient or relatives. The fear of patients complaining that an error has occurred due to negligence. Nursing administration focuses on the individual rather than looking at the systems as a potential cause of the error. Too much emphasis is placed on MEs as a measure of the quality of nursing care provided. There is no support for the nurse when an error occurs. The lack of an administrative system. Lack of instruction in the hospital on the definition of errors resulting from giving drugs.
Rutledge et al. (2018) [36]	United States	The study’s purpose was to report ME reporting barriers among hospital nurses and to determine the validity and reliability of an existing MERB questionnaire.	Cross-sectional study design	359 nurses	Extra time involved in documenting ME. The system for forms used to report ME is long and time-consuming. Fear of liability or lawsuits. Fear of being blamed. Fear of disciplinary action.
Dirik et al. (2019) [39]	Turkey	To investigate hospital nurses’ involvement in the identification and reporting of MEs in Turkey.	Cross-sectional study design	135 nurses	Afraid/hesitant to be seen as incompetent by peers. Afraid/hesitant of being punished by managers. Unaware a mistake has been made. They believe that reporting is unnecessary if the patient was not harmed. Afraid/hesitant of a negative reaction from the patient or relatives. No positive feedback was given to the person who reports the error. Considering the error is not serious enough to report. Afraid/hesitant of physicians’ negative reactions. Fear of losing his/her job. Lack of a clear definition of ME in the institution. Lack of training for nurses about medication errors. Unaware of an error reporting form/process. Completion of error reporting form takes too long.
Hammoudi et al. (2018) [31]	Saudi Arabia	To assess the factors contributing to the occurrence and reporting of MEs from the nurse’s perspective.	Cross-sectional study design	367 nurses.	Nurses do not agree with the hospital’s definition of a ME. ME is not clearly defined. Nurses did not see the error to be important enough to report. Filling out an incident report for a medication error takes too much time. Too much emphasis is placed on MEs as a measure of the quality of nursing care. Nursing administration focuses on the individual rather than looking at the systems as a potential cause of the error. Nurses fear adverse consequences from reporting MEs.
Amrollahi, et al. (2017) [29]	Iran	To determine nurses’ perspectives on the reasons behind MEs and the barriers to error reporting	Cross-sectional study design	213 nurses	Fear over the negative effects of error reporting on salaries. Unfair supervisory reactions are disproportionate to error seriousness. Forgetting to report MEs. Fear over the negative effects on annual staff evaluation. Fear of blame from the supervisor. Unclear definition of MEs.
Albukhodaah, et al. (2016) [13]	Saudi Arabia	To identify potential barriers or challenges that may influence reporting of MAEs among nurses in Saudi Arabia	Mixed method design (qualitative and quantitative)	366 nurses	Fear of punishment from the administration. The administration focuses on the individual, not the system. No feedback after reporting MEs. Nurses are concerned about patients or families developing a negative attitude towards them with a loss of confidence in their nursing abilities. Nurses are concerned about facing lawsuits or legal action by patients or family. Nurses felt they might be seen as criminals when they report MEs.

During the data analysis, two major themes and five subthemes regarding barriers to MAEs reporting emerged. The two major themes included organisational barriers and professional and behaviour-related barriers to reporting ME as shown in Table 3.

**Table 3:** Themes generated from data analysis

Main themes	Subthemes	Free codes
Organisational barriers	Reporting system [31–34, 36, 38, 39]	No proper reporting system, consumed time, long forms for reporting, heavy workload
	Definition of ME [28–31, 37, 39]	Unclear definition, no standard definition
	Management behaviour [13, 28–32, 34–39]	Punitive actions, negative response, no feedback after reporting, targeting the individual than the system, blame culture, lack of confidentiality
Professional and behavioural barriers	Personal reasons/ lawsuit [27, 29, 30, 32, 33, 35, 36, 39]	Fear of reporting, being stigmatized, fear of legal action, forgetfulness, fear of being called incompetent or inadequate
	Knowledge of error [31, 33, 35, 38, 39]	Inadequate knowledge on what constitutes error, unable to recognize an error, unaware of error occurrence

**Legend:** Table 3 shows the barriers to MAE reporting by nurses

### Organisational barriers

Organisational barriers were categorized into three sub-themes of barriers to ME reporting: reporting system, definitions of MEs, and management behaviour. The sub-themes are described below in more detail.

#### Reporting system

The researchers identified in the studies that there was no clear or proper ME reporting system [38] therefore making the process of reporting cumbersome, especially the use of the medication incident reporting form which served as a major barrier to reporting MEs [33]. Some studies documented that ME reporting consumed much time [31, 34, 36, 38, 39], whiles Dyab et al. [32] reported lack of time, tiredness, and heavy workload as barriers to reporting MEs. Rutledge et al. [36] revealed that the forms used to report MEs are long which posed as a barrier to reporting MEs.

#### Definitions of medication error

It was indicated in some studies that because there was no precise definition of ME within the hospital [28, 29, 31, 37, 39], there were disagreements regarding the definition of ME and what should constitute a reporting event [30, 31, 37, 39].

#### Management behaviour

Several studies revealed that reporting MAEs may result in punitive actions by management or negative consequence [13, 27, 29–32, 34, 36, 37], thereby creating fear among nurses [27, 32, 34, 36, 37]. Also, a negative response from the hospital administration was identified by Shahzadi et al. [38] as a key deterrent to reporting MEs by nurses. Nurses indicated in several studies that they were not given feedback after reporting MAEs [13, 27, 30, 32, 34, 39] which contributed to underreporting of MEs. The researchers also observed that the nursing administration focuses on the individual rather than using the systems approach to solve the problems [13, 27, 28, 30, 31] which served as a major barrier to reporting MEs. Nurses indicated that too much emphasis is placed on MEs as a measure of the quality of nursing care [28, 30, 31] therefore impeding error reporting. Nurse’s feared of being blamed by management [31, 32, 35, 36] if they reported MEs and this served a barrier. Lack of confidentiality in management was also a barrier to reporting MEs [32].

### Professional and behavioural barriers

Under the professional behavioural barriers, two sub-themes were identified: personal reasons, and knowledge of error. The sub-themes are described below in more detail.

#### Personal reasons/lawsuit

Personal reasons such as criticism from colleagues or other professionals was a barrier to ME reporting [34, 39] because they felt they would be embarrassed or discriminated against if they reported MAEs [32]. Nurses personally felt they could be blamed [36] if something negative happened to the patient [30] so they were not encouraged to report MEs. Nurses feared that reporting MEs would negatively impact their job records [32] or they might lose their job [33, 39] which served as an impediment to reporting MEs. A tag on their professional identity or fear of being labelled as incompetent and an inadequate nurse [35] was also identified as a barrier to ME reporting. One major key factor impeding ME reporting in some studies was the fear of legal actions against nurses by patients or their families [13, 27, 34, 36]. Forgetting to report ME was another individual barrier to reporting ME [29].

#### Knowledge of medication error

Inadequate knowledge of nurses about what constitutes ME [33] led to underreporting. Nurses did not see the gravity of the ME to warrant reporting [31, 33, 38]. The inability of nurses to identify that an error has occurred hindered reporting of MEs [33, 35, 38]. MAEs that occurred without patient harm did not warrant reporting [35]. Unawareness of the occurrence of MEs [39] also led to nurses not reporting MEs.

## Discussion

This study reviewed and synthesized results on barriers to reporting MAEs among nurses. The major barriers include [1] organisational, and [2] professional and behavioural barriers. These are results of studies from different countries ranging from low- middle-, and high-income countries. Therefore, the findings from this review can be vital for the global healthcare communities to improve patient safety as it remains one of the biggest global challenges in healthcare. Most of the studies included in this review were rated as strong, and moderate inferring that the evidence produced from this review has a strong and justified conclusion, meaning that implications can be drawn for nursing research and practice. Also, this study aligns with the WHO `Global Patient Safety Challenge’ emphasizing the promotion and improvement of patient safety actions to reduce severe, preventable medication-related harm by 50% in the next five years [7]. To develop an effective and robust intervention to improve patient safety, MAE reporting is essential and grounded through the identification of barriers based on the consideration of behavioural change theories [40]. This information garnered from the key clinical practicing professionals will go a long way to inform policy, healthcare organisations, and other stakeholders on measures to mitigate these barriers and improve patient safety within our healthcare settings across the globe.

The current review found organisational barriers to be the most prominent barrier for nurses not reporting/underreporting MAEs. Barriers such as lack of proper reporting systems, no clear definition of MAEs, and punitive actions against nurses after reporting MAE were identified as organisational barriers to reporting MAEs. Many MAEs go unreported due to the lack of reporting systems or lack of proper reporting systems [41]. It is imperative to know that if there are no proper reporting systems for MAEs in health facilities, then nurses will find it difficult to duly report errors. Therefore, an established system for reporting MEs in hospitals is important to improving patient safety measures. Established good reporting systems are avenues for collecting vital and sufficient information about MAEs from different reporters [41]. This information gathered will help reporters understand the factors that influence errors and will therefore subsequently help to prevent their recurrence [41]. Generally, it is observed that nurses’ failure to report MEs is related to the aftermath consequences they may suffer after reporting depending on the severity of the incidence of injury [42]. It is observed that some health practitioners fail to report errors due to the intense follow-up investigations on persons that commit these errors rather than the system. Nurses believe that reporting errors negatively impact their future job appraisals and professional development due to the punitive actions taken against them. Non-punitive actions against health care professionals who report errors are recommended to improve patient safety care [22, 42, 43]. Several studies have documented that health professionals who are rewarded and motivated for reporting errors during healthcare are encouraged to further improve on their reporting behaviour which subsequently improves patient safety in the organisation [22, 43]. It is also noted that many organisations have been challenged to provide an environment that is free and safe to admit errors and to understand why they occur void of reprisal and punishment [44].

Criminal prosecution of healthcare professionals in the line of duty remains an astonishing event. Over the years the number of healthcare professionals facing legal actions continues to increase [45], indicating that healthcare professionals should take strong actions to address these issues. This review revealed that nurses were afraid to report MEs due to possible lawsuits and lack of confidentiality or anonymity in the reporting system. When designing a reporting system, anonymity should be considered to be an important factor [22] because an anonymous system means a non-punitive reporting culture [46] and no traceable follow-up procedures after reporting medication incidents [47]. An anonymous medication error reporting system could help to overcome these barriers of not reporting. A study by Hurley and Berghahn [45] reported two cases in which nurses were prosecuted for criminal negligence related to MAEs. In order to enhance ME reporting, it is imperative to address systemic issues and problems within the institutions but not the individual.

Inadequate knowledge of nurses about what constitutes ME [33] and their inability to identify ME necessitating error reporting [33, 35, 38] were barriers to error reporting. Nurses’ knowledge of ME reporting is an important factor that determines the success of the medication reporting system [32]. It has been recommended that a blend of formal educational seminars (patient safety lectures), and informal educational sessions (lunchtime educational sessions or an online tutorial on using a new reporting system) could improve error reporting [43]. Therefore, organisations should develop interventional educational programs tailored toward continuous professional education of nurses on MEs reporting systems to improve medication safety. As some studies have found a strong correlation between healthcare workers attending patient safety training workshops and the increased rate of error reporting [43, 48].

### Limitations

This review had several limitations. First, 12 of the studies included in this review were clustered in Asia, one each in the United States and Ethiopia. These countries captured in this review are not sufficient for the entire world. Second, this study included only published articles in English which might have excluded relevant evidence published in other languages. Third, authors may have unintentionally omitted relevant studies from this review although extensive database and hand searches were conducted. Finally, the review focused on only nurses, and this might have caused the loss of some vital information on studies conducted among other health care professionals such as pharmacists and doctors. That notwithstanding nurses are the final point of drug administration so therefore, this study provides a comprehensive insight into barriers to reporting MAEs among nurses. These findings could help inform policy decision-making in order to improve patient safety through reporting MAEs.

## Conclusion

Providing an enabling environment void of punitive measures and blame culture is imperative for nurses to report MEs. The institutionalisation of a proper reporting system for ME reporting provides an avenue to gather data for root cause analysis of errors. This will further enhance a systems approach in dealing with the problems and issues with MEs without focusing on the individual. To minimise the burden on nurses reporting MEs, an effective, non-time consuming, and the uncomplicated anonymous system is required. An open feedback system for motivating or rewarding nurses for reporting MEs is imperative and will therefore increase the rate of MAE reporting. Policymakers, managers, and Nurses should agree on a uniform definition of what constitutes ME to enhance nurses’ ability to report.

## Supplementary Information

**Additional file 1.** Search strategy.

## Data Availability

The datasets used and/or analysed during the current study are available from the corresponding author on reasonable request.

## Abbreviations

CINAHL: Cumulative Index to Nursing and Allied Health Literature
MMAT: Mixed Methods Appraisal Tool
US: United States
IOM: Institute of Medicine
WHO: World Health Organisation
ME: Medication Error
MAE: Medication Administration Error
PRISMA: Preferred Reporting Items for Systematic reviews and Meta-Analysis guidelines
