# Supplementary material for: Improving patient safety through identifying barriers to reporting medication administration errors among nurses: an integrative review
Source: BMC Health Serv Res. 2021 Oct 25;21:1156. doi: 10.1186/s12913-021-07187-5 (PMC8547021; doi:10.1186/s12913-021-07187-5)
Supplement: Supplementary file 1 — Additional file 1. Search strategy. [file 12913_2021_7187_MOESM1_ESM.docx]

**Search strategy**

**Date: 2016 January -2020 December**

| **No** | **Data source** | **Search strategy** | **results** |
| --- | --- | --- | --- |
| 1 | PubMed | “medication error*” OR “medication administration error*” OR “drug error*” OR “drug administration error*” OR “medication error report*" OR “drug error report*” | 3,278 |
| 2 | Web of Science | “medication error*” OR “medication administration error*” OR “drug error*” OR “drug administration error*” OR “medication error report*" OR “drug error report*” | 3,724 |
|  | EMBASE | “medication error*” OR “medication administration error*” OR “drug error*” OR “drug administration error*” OR “medication error report*" OR “drug error report*” | 1,034 |
|  | CINAHL | “medication error*” OR “medication administration error*” OR “drug error*” OR “drug administration error*” OR “medication error report*" OR “drug error report*” | 2,893 |
|  |  |  | 10,925 |
